# The Medical Necessity of Orthodontic Care: A Qualitative Study

**DOI:** 10.1177/23800844251355270

**Published:** 2025-07-17

**Authors:** D. Richmond, H. Benzian, J. Daskalogiannakis, A. Holden, C. Quiñonez

**Affiliations:** 1 Graduate Orthodontics, Faculty of Dentistry, University of Toronto, Toronto, ON, Canada; 2 WHO Collaborating Center Quality Improvement & Evidence-Based Dentistry, New York University College of Dentistry, New York, NY, USA; 3 Sydney Dental Hospital and Oral Health Services, Sydney Local Health District, Surry Hills, NSW, Australia; 4 Schulich School of Medicine & Dentistry, Western University, London, ON, Canada

**Keywords:** delivery of health care, health policy, health services accessibility, health priorities, needs assessment, public health dentistry

## Abstract

**Introduction:**

As global momentum builds for universal health coverage (UHC), it is unclear whether orthodontic care should be included in UHC packages. The concept of medically necessary orthodontic care (MNOC) and its criteria thus have far-reaching implications for priority setting and resource allocation in public and private oral health care programs.

**Objective:**

To identify factors that contribute to the determination of MNOC based on perspectives from leaders of dental professional organizations, academics, clinicians, funders, patient advocates, and patients from 7 countries: Canada, United States, Germany, Greece, United Kingdom, Switzerland, and Australia.

**Methods:**

A qualitative description design was used with semi-structured virtual interviews conducted via Zoom from November 2021 to August 2022. Interviews were transcribed verbatim, coded, and analyzed for themes.

**Results:**

Sixteen interviews were conducted. Participants described their concept of MNOC through 4 interrelated categories: (1) dental factors including dental health, the goals of treatment, and methods of needs assessment; (2) medical factors including the meaning of medical necessity, systemic health considerations, and treatment of craniofacial anomalies; (3) psychosocial factors including societal standards of beauty, social functioning, and mental health; and (4) funding factors including resource allocation considerations and the goals of funding.

**Conclusion:**

The diversity of factors identified highlights the complex interplay between the dental profession, funders of care, society, and individual patients in understanding MNOC. Given this complexity, MNOC is arguably not amenable to a concise definition or list of criteria. Instead, a decision-making process that incorporates key actor perspectives can enhance transparency, fairness, and accountability in priority setting and resource allocation as related to MNOC and medically necessary oral health care more broadly. This approach would ensure coverage for those with demonstrated need in the context of health, well-being, and quality of life.

**Knowledge Transfer Statement:**

This study provides critical insights into the dental, medical, psychosocial, and funding factors that influence the meaning of medically necessary orthodontic care (MNOC) from the perspectives of key actors in 7 high-income countries. The findings reveal that MNOC cannot be defined by a simple set of criteria. Instead, determinations of MNOC should be made through a decision-making process that incorporates a wide array of viewpoints. This approach ensures transparent and fair resource allocation, improving access to essential orthodontic services, thereby enhancing patient health and well-being.

## Introduction

With growing global support for the inclusion of oral health care within universal health coverage (UHC), the status of orthodontic care (OC) in such a context remains an open question. Is OC “medically necessary” (MN) or simply a consumer demand? The concept of “medically necessary orthodontic care” (MNOC) and its criteria thus have implications for priority setting and resource allocation in public and private oral health care programs.

The American Association of Orthodontists (AAO) defines MNOC as “orthodontic services to prevent, diagnose, minimize, alleviate, correct, or resolve a malocclusion (including craniofacial abnormalities and traumatic or pathologic anatomical deviations) that cause pain or suffering, physical deformity, significant malfunction, aggravates a condition, or results in further injury or infirmity” (AAO 2019). In addition, the AAO sets stringent auto-qualifiers for MNOC, such as an overjet of 9 mm or greater or an impinging overbite with documented occlusal contact into the opposing soft tissue, which results in only a small percentage of cases qualifying as MN. Malocclusion severity indices have also been developed to determine MNOC (Borzabadi-Farahani 2011). Used worldwide, these indices are important tools in priority setting and resource allocation for the public funding of OC (Shaw et al. 1995; Borzabadi-Farahani 2011).

Despite the widespread use of MN and synonymous terms (e.g., “essential”) in health care legislation and policy, these terms are notoriously ambiguous, creating controversy over their interpretation (Charles et al. 1997). In Canada, for example, the meaning of MN is hinted at in health care legislation rather than explicitly defined (Health Canada 2024), doing little to distinguish necessary from unnecessary services. Similar issues arise in orthodontics. While definitions of MNOC capture the scope of conditions that OC should address, they do little to establish a threshold for what is MN or essential. Likewise, the AAO has not recognized any one index as valid for measuring orthodontic treatment need (Shaw et al. 1995; AAO 2016). Finally, the societal importance of dental appearance cannot be ignored. While OC may not be a matter of life and death, its role in enhancing social interactions, employability, and preventing stigmatization, all of which contribute to overall health and well-being, should be considered in priority setting and resource allocation discussions, adding another layer of complexity to the issue (Singhal et al. 2013; Khalid and Quiñonez 2015).

It is challenging to determine how prominently OC should feature in UHC packages, if at all. While some high-income nations offer partial coverage for OC to certain populations under certain circumstances, these circumstances vary by nation and even subnationally. Determining a clinically and policy relevant understanding of MNOC can thus assist policymakers in setting priorities around the allocation of scarce oral health care resources. Therefore, this study aimed to identify factors that contribute to the determination of MNOC by analyzing perspectives from leaders of dental professional organizations, academics, clinicians, funders, patient advocates, and patients.

## Materials and Methods

### Approach

The University of Toronto Health Sciences Research Ethics Board approved the study (protocol 00041649). The meaning of MNOC was explored through a qualitative description (QD) methodology using individual open-ended, semi-structured interviews (Sandelowski 2000; Neergaard et al. 2009). QD aids in determining accurate descriptions of phenomena about which little is known to answer questions of relevance to practitioners and policymakers (Sandelowski 2000).

### Researcher Reflexivity

In qualitative research, it is important to recognize how personal, cultural, and professional experiences shape the interpretation of findings (Braun and Clarke 2006). Reflexivity allows the researcher to examine how their background influences the research process and outcomes. This passage reflects on my (D.R.) biases, values, and experiences with the medical necessity of OC, providing transparency and maintaining the study’s integrity.

As both a patient and a health care provider, my experiences shaped my perspective on MNOC. As an adolescent, orthodontic treatment boosted my confidence and self-esteem. My dental education and clinical practice reinforced my belief in the value of care, leading me to pursue specialty training in orthodontics. As an orthodontic resident conducting this research and now as a licensed orthodontist, I remain open to the idea that OC may not always be MN but rather a wish-fulfilling treatment for those who seek it. In addition, while I conducted the data collection and most of the analysis independently, my thesis committee members and co-authors—experts in clinical orthodontics (J.D.) and policy and public health (H.B., A.H., C.Q.)—also guided the study design and data interpretation. They feel there is room for improvement in current systems for determining MNOC, particularly as medicine transitions from a purely biomedical to a more biopsychosocial approach. While these perspectives, especially their focus on policy and public health, influenced my approach to data collection, analysis, and interpretation, I remained committed to maintaining analytical neutrality.

By acknowledging these experiences and striving to minimize bias, I aimed to conduct this research objectively, ensuring my views did not unduly shape the study’s methods or outcomes. Ultimately, I hope this research will inform open discussions with future patients, policymakers, and funders about the necessity of OC as it relates to priority setting and resource allocation in public and private oral health care programs.

### Sampling

Individuals who could provide insights into MNOC were purposefully sampled through a researcher-developed contact list. The primary researcher compiled this list through team meetings with coauthors, who identified potential participants with relevant knowledge of MNOC from their professional networks. To ensure maximum variation, participants were selected to represent a range of geographic regions (North America, Europe, and Australia) and professional roles (clinicians, academics, policymakers, funders, advocates, and patients). This approach allowed the study to capture a broad and diverse set of perspectives on MNOC.

Participants were included if they had direct experience with MNOC in policy development, academic research, orthodontic practice, service funding, social services delivery, or patient advocacy or if they were patients themselves. Exclusion criteria applied to individuals with no direct involvement in MNOC-related fields or those unable to participate in a virtual, English-language interview.

Recruitment commenced on November 10, 2021, with 45 potential participants contacted via email to describe the study and their potential involvement. Due to the limited pool of potential participants, all coauthors were invited to participate as they are experts in relevant fields and have had various involvements with MNOC, whether clinically or from a policy standpoint. Those who expressed interest in participating were taken through an informed consent process, which included signing and returning a consent form via email. Snowball sampling was also used to identify other individuals who could provide insights into MNOC. Recruitment continued until data saturation was reached, defined as the point at which additional data did not further improve understanding of the phenomenon under study (Bedos et al. 2009). The recruitment period concluded on June 14, 2022.

### Data Collection

Interviews were guided by a questionnaire developed from a narrative literature review on MNOC-related topics. The interviews were conducted online using Zoom (Zoom Video Communications) by the primary researcher during his second and third year of orthodontic residency.

Each interview began with a brief explanation of the research problem and objectives, followed by verbal confirmation that the recording had started. Questions from the interview guide were asked, although the order in which they were asked varied based on the flow of the conversation. The interview guide covered topics such as participants’ personal experiences with orthodontics, their views on the medical necessity of OC, key factors in determining MNOC, funding mechanisms, patient preferences, and the ethical implications of accessibility. All interviews were recorded and transcribed verbatim. Interviews were conducted between November 30, 2021, and August 19, 2022, with some participants scheduling interviews after the recruitment period had closed.

This project had an emergent design, meaning that the research process evolved as data collection progressed, with adjustments made based on emerging insights (Creswell 2013). As the study unfolded, interview questions were adapted in response to the data. For instance, a question about funding allocation was added, and the order of questions was adjusted to allow for a more natural flow of conversation. In addition, the primary researcher became more adept at asking probing questions, informed by insights from earlier interviews. These adaptations ensured the research process remained flexible and responsive to participants’ perspectives.

### Data Analysis

The primary researcher conducted a reflexive thematic analysis based on the 6-phase approach by Braun and Clarke, which enabled in-depth exploration of participant perspectives and generated valuable insights, particularly for informing policy development (Braun and Clarke 2006, 2021a). Phase 1: Familiarization involved reading through anonymized transcripts to become familiar with the data and making notes regarding potential themes from each interview. In Phase 2: Coding, transcripts were imported into NVivo 12 (QSR International; Melbourne, Australia) and statements were identified and coded. Codes were defined as concise summaries of what the participant said, without interpretation or assumptions, to minimize researcher bias. Initial coding was done line by line to ensure all relevant data were captured. Overlapping codes were combined, and irrelevant codes were excluded. In Phase 3: Generating Initial Themes, clusters of codes were organized into broader, nonoverlapping themes, capturing patterns about the data in relation to the research question. Themes were reviewed in Phase 4: Theme Development and Review, in which the primary researcher re-read coded extracts and considered the themes in relation to the research question to ensure their relevance and clarity. In Phase 5: Refining, Defining, and Naming Themes, the primary researcher refined the themes, redefined them where necessary, and named them to reflect the essence of participants’ experiences/perspectives. In Phase 6: Writing, the themes and subthemes were organized into a table to present an overview of the results, and transcript extracts were selected to support each subtheme. The process was iterative, with continuous review and refinement to ensure the final analysis best represented the data.

Data saturation was assessed following Braun and Clarke’s approach, which recommends determining the final sample size based on the adequacy and richness of the data for answering the research question (Braun and Clarke 2021b). Saturation was reached after analyzing the 10th interview, as no new codes or themes emerged and sufficient information had been gathered to address the research question. However, the decision was made to continue collecting data beyond the point of saturation to include perspectives of additional key actors whose voices had not yet been heard, resulting in a final sample size of 16 participants. The final themes and subthemes were organized into a table, with extracts from the transcript selected to illustrate and support each subtheme.

### Rigor and Validity

To promote rigor, several strategies were implemented to uphold methodological integrity and faithfully represent participants’ experiences/perspectives while minimizing researcher bias. Participants were assured of confidentiality to foster open dialogue, allowing them to guide the conversation and establish rapport with the primary researcher (Milne and Oberle 2005). Flexibility in the interview guide encouraged natural conversations, ensuring all relevant topics were addressed. Probing questions and clarifications facilitated a comprehensive understanding of participants’ viewpoints, minimizing the potential for researcher bias in shaping the conversation (Milne and Oberle 2005). Interviews were transcribed verbatim, with nonverbal cues noted from video recordings. Demographic surveys and questions about orthodontic experiences provided additional context to the interviews (Milne and Oberle 2005). Reflexive thematic analysis was applied to the data, ensuring a high-quality interpretation of participants’ experiences/perspectives and a systematic minimization of bias throughout the process (Braun and Clarke 2021a).

To heighten the validity of the findings, both internal and external strategies were employed. Internal validity was maintained through reflexivity and peer review (Milne and Oberle 2005). Reflexivity enabled the primary researcher to consistently reflect on personal biases, maximizing interpretations of the data that aligned with participants’ experiences. Peer review by committee members provided additional layers of oversight, ensuring the research addressed relevant issues and that any researcher bias was further minimized. For external validity, purposeful sampling ensured that a broad range of experiences/perspectives was captured, enhancing the applicability of the findings (Milne and Oberle 2005; Creswell 2013). Triangulation was achieved by incorporating various viewpoints (Creswell 2013). Finally, the study’s findings were compared with existing literature, confirming their relevance and consistency with broader discussions in the field (Braun and Clarke 2006).

## Results

Sixteen participants (14 male, 2 female) from 7 high-income nations were interviewed (Table 1). Participants represented a variety of roles and were involved in a range of activities related to OC, such as clinical care, policy development, funding decisions, and lived experience (Table 2). Interviews averaged 51 min, with durations ranging from 17 to 104 min. Thematic analysis resulted in 11 themes and 25 subthemes relevant to determining MNOC, which were organized into 4 interrelated categories: dental, medical, psychosocial, and funding factors (Table 3).

**Table 1.** Geographic Distribution of Participants.

Country	No. of Participants
Canada	7
United States	2
Germany	2
Greece	2
United Kingdom	1
Switzerland	1
Australia	1

**Table 2.** Stakeholder Categories Represented by Participants.

Job Description	No. of Participants
Academia	7
Social services expert	3
Clinical expert	2
Funder	1
Leader of orthodontic organization	1
Public policy expert	1
Patient	1

**Table 3.** Factors Influencing the Meaning of MNOC Identified through Thematic Analysis.

Dental	Medical	Psychosocial	Funding
(A) Impact on dental health 1. Orthodontic care can improve current issues 2. Orthodontic care may reduce risk of future issues (B) Aims of orthodontics 3. Goals of orthodontic care are unclear 4. Fallacies of ideal occlusion 5. Patients want chief complaint addressed (C) Assessment of orthodontic need 6. Indices determine the worst cases 7. Auto-qualifiers detect the extremes 8. Experts believe their opinion can define necessity	(D) Interpretation of medical necessity 9. The meaning of medical necessity is elusive (E) Impact on systemic health 10. Survival is not affected by orthodontic care 11. Ability to function may be affected by orthodontic care (F) Orthodontics for those with medical conditions 12. Necessary treatment determined by a medical diagnosis 13. Different aims of treatment for craniofacial anomalies	(G) Complying with societal standards of beauty 14. Pressure from dental industry to have a perfect smile 15. Pressure from society to have a pleasing smile (H) Social functioning 16. Improved social success with pleasing smile esthetics 17. Negative social interactions stemming from malocclusion (I) Mental health 18. Improving subjective well-being through orthodontic care 19. Addressing mental illness through orthodontic care	(J) Resource allocation 20. Available resources affect the threshold for MNOC 21. Decision-making processes affect the focus of MNOC 22. Orthodontics is not a priority compared with other health care concerns (K) Goals of funding 23. Funding decisions depend on the values of society 24. Context affects funding decisions 25. Cost-effectiveness is a challenge for orthodontic care

MNOC, medically necessary orthodontic care.

### Dental Factors

Most participants believed that OC should be considered MN if it leads to improvements in dental health or reduces the risk of future disease. However, others acknowledged that this may hold true for only a minority of cases or those most severe in nature.

Some felt that the goals of OC are unclear, making it challenging to determine need, especially since patients often seek OC to have esthetic complaints addressed, irrespective of the treatment goals defined by the specialty or funders. One participant explained that a definition of MNOC might change based on whether the goal of treatment is to “get a perfect occlusion,” “achieve the chief complaint of the patient,” or “achieve certain metrics defined by a funder” (participant 15, male, public policy expert); thus, without a clear understanding of the goals of OC, there is a risk of turning “things that are unesthetic into a disease [leading to] the medicalization of things that didn’t need to be medical problems” (participant 16, male, social services expert).

Most participants had mixed feelings regarding the ability of indices and auto-qualifiers to assess need. Perceived benefits included objectivity, fairness, and ability to classify severe malocclusions. Drawbacks included arbitrary thresholds, too much focus on dental and cephalometric measurements, and limited emphasis on patient-relevant outcomes. One participant noted that auto-qualifiers are “a great way to start with making this a more rational decision” (participant 16, male, social services expert); however, “you look at their auto-qualifiers and it’s strictly what the ruler is telling you. There’s nothing here about how does it impact their social interactions, their confidence, self-esteem” (participant 16, male, social services expert).

### Medical Factors

Before MNOC can be defined, participants emphasized the need for consensus on the meaning of medical necessity. Interpretations of medical necessity included services that conveyed health benefits and improvements in function and quality of life. According to one participant, the meaning of MNOC “depends on how you define medical treatment, because the older philosophy [was] just biological treatment. Now, it’s a social biological philosophy. You have psychological needs, [which are] considered an important part of well-being” (participant 6, male, academia). Importantly, some participants questioned the scientific basis for linking orthodontic treatment to medical necessity. For instance, OC should be considered MN when it improves one’s ability to “speak, talk, and eat” (participant 9, male, social services expert), but “there’s no scientific evidence to relate occlusion to [what is] medically necessary” (participant 3, male, academia).

All participants agreed that those with craniofacial anomalies have the highest need for OC. In these cases, the goals of treatment are clearer. One participant highlighted that “the presence of a craniofacial anomaly is a bit easier to define as medical necessity, because the malocclusion is the result of the deformity” (participant 5, male, academia). For these patients, the goals of treatment are more straightforward: restore form, function, and esthetics to as close to normal as possible and, in turn, improve aspects such as confidence and self-esteem. As one participant put it, “we’re not looking for [ideal] cases here. If the kid sort of looks [more like everyone else], then we’ve done a big job . . . I don’t think anybody’s going to get too excited if his mesiobuccal cusp is not in the mesiobuccal groove” (participant 3, male, academia).

### Psychosocial Factors

Most participants felt that societal standards of beauty force a reassessment of MNOC. Not having a “perfect smile” may lead to negative psychosocial outcomes, and thus OC can be viewed as MN in this context. For instance, there is “pressure put on everyone to comply with a certain standard of beauty” (participant 16, male, social services expert) and “clearly in society, how well you do is not just based on how hard you work” (participant 12, male, social services expert). The way you look “has an impact on what your outcomes are likely to be, whether that’s occupational outcomes [or] relationship outcomes” (participant 12, male, social services expert).

Participants also discussed the role of OC in subjective well-being, particularly in mental health. OC was described as transformative, capable of producing a “beaming young person who has a lot more confidence” (participant 9, male, social services expert). In contrast, those with malocclusion “carry the stigma of teeth that are not looking good, even though this is not a functional reason. But it’s functional in a mental health sort of way” (participant 13, male, clinical expert).

### Funding Factors

All participants recognized that MNOC is a tool for priority setting and resource allocation. Therefore, some argued that the boundaries of MNOC can fluctuate based on the availability of resources. If a “funder decides to make a value judgment and say we really want to fund this, maybe the threshold [for MNOC] can be loosened . . . but what if you’re dealing with a real scarcity of resources? Maybe that threshold gets tighter” (participant 15, male, public policy expert). Others argued that the concept of MNOC is fixed, yet its relative prioritization can change based on other needs inside and outside of dentistry, as well as a nation’s socioeconomic and professional context. For example, “if you were going to divert funding into anything it [should] be management of caries . . . in young people” (participant 12, male, social services expert), and in “childhood cancer versus lower incisor irregularity, where do you put your money?” (participant 4, male, academia). Similarly, “need [is] very different when you move to low- and middle-income countries because there it is very clearly linked to availability and affordability. If you live in a country that has 12 dentists for the population of 20 million, orthodontics is nowhere, nothing that you can even wish for” (participant 16, male, social services expert).

Some participants raised concerns about cost-effectiveness, citing the lack of robust evidence supporting the benefits of OC. For instance, “you’ve got to look at treatment in terms of its overall cost-benefit, but in order to do that, then you’ve got to measure the risks and benefits of orthodontic treatment, which is a sort of a real Pandora’s box to get into” (participant 4, male, academia).

## Discussion

This study used a QD methodology to gain an in-depth understanding of key actor perspectives on MNOC, providing valuable insights for practitioners and policymakers shaping oral health care systems (Sandelowski 2000; Neergaard et al. 2009). Participants highlighted 4 categories of interrelated factors relevant to discerning MNOC: dental, medical, psychosocial, and funding. Participants believed that for certain patients, there are dental and medical benefits to providing OC, speaking to the most biomedical sense of MNOC. Many also felt that OC provides psychosocial benefits, and this should be captured in a definition of MNOC, reflecting evolving conceptions of health. Funding considerations force a line to be drawn, as resource availability will inevitably influence how the boundaries of MNOC are defined.

### MNOC in Current Practice

These results highlight important questions in current approaches to MNOC. In public programs, the concept of MNOC functions as an aid for coverage decisions. Indices are used (with or without auto-qualifiers) to operationalize MNOC, relying on clinical measurements recorded by care providers (Minick et al. 2017; Camacho et al. 2022). These indices, which serve as a proxy for MNOC, were designed to facilitate access to care for those with the greatest need (Shaw et al. 1995). Nevertheless, as participants pointed out, this approach is not free from scrutiny and controversy. For instance, indices created based on the opinions of orthodontists and others in one location may not be generalizable elsewhere (Younis et al. 1997). Perceptions of need also change, potentially reducing the relevance of an index assessment over time. In addition, thresholds for MNOC are influenced by public policy and financial constraints (Borzabadi-Farahani 2011; Minick et al. 2017). In the United States, for example, thresholds for MNOC are adjusted annually based on resource availability, exposing the illusion of scientific objectivity in indices that ultimately bend to economic and political pressures (Minick et al. 2017).

Perhaps surprisingly, it appears that the very aims of OC are unclear. Traditionally, OC was conceptualized as improving oral health and function; however, this concept has since faded. Ideal occlusion is no longer seen as an absolute state that confers optimal health but more of a professional ideal that clinicians strive for (Ackerman 2010). Although participants felt OC can benefit oral health, they acknowledged that evidence is lacking in the association between malocclusion and oral or general health. Decades ago, Shaw found that only increased overjet, traumatic deep bites, and impacted teeth have negative effects on oral health (Shaw et al. 1991). A more recent systematic review examining the connections between malocclusion, orthodontic treatment, and oral health concluded that among all outcomes, only a reduced risk of trauma following overjet correction was supported by the evidence (Macey et al. 2020). Taken together, this suggests that the concept of MNOC must consider factors beyond purported improvements to oral and systemic health.

Current priority setting and resource allocation approaches privilege care for those with craniofacial anomalies (Borzabadi-Farahani 2011). There was unanimous agreement among participants that OC for these populations should be considered MN. Their classification as MN stems from functional, reconstructive, and esthetic considerations. Whether patients are identified through auto-qualifiers, a high index score, or a separate overriding system based on medical diagnosis, most funders appear sensitive to these conditions, as confirmed by both participants and the literature (Borzabadi-Farahani 2011; AAO 2019).

### What’s Missing?

MNOC in current practice lacks emphasis on patient-determined need (Gherunpong et al. 2006; Camacho et al. 2022). Since malocclusion does not pose a biomedical health risk yet offers psychological benefits, experts recommend prioritizing patient perspectives in deciding the need for treatment (O’Brien et al. 1998; Camacho et al. 2022; Gherunpong et al. 2006). Participants spoke to this by suggesting that a patient’s chief complaint, which is influenced by society’s ever-changing beauty standards, should be given weight when discerning MNOC. Indeed, patients are considered the best judges of their oral health–related quality of life and the impact of malocclusion on their daily life (Tsakos 2008).

In this regard, participants’ suggestion that most who seek OC are driven by esthetic reasons aligns well with available literature (Kiyak 2008; Wędrychowska-Szulc and Syryńska 2010; Prado et al. 2022). Patients are concerned with esthetic issues such as anterior crowding, spacing between incisors, and/or increased overjet (Dimberg et al. 2015). The associations between OC and improved oral health–related quality of life are also well documented. Many studies have shown improvements in satisfaction with one’s smile, interpersonal relationships, and psychological well-being (Liu et al. 2009; Jung 2010; Dimberg et al. 2015; Alrashed and Alqerban 2021). Untreated malocclusion has been associated with hiding one’s smile, bullying, reduced social activities, and even academic underperformance and absenteeism (Basha et al. 2016; Silva et al. 2022; DiBiase et al. 2024). Thus, OC for esthetic concerns can be justified on grounds that transcend mere vanity, reflecting its recognized impact on overall well-being.

The literature also shows that malocclusion fluctuates in importance given individual context. People’s feelings toward the need for OC are influenced by gender, age, social media use, socioeconomic status, insurance coverage, peer and social group influence, and perceptions of facial esthetics (Germa et al. 2010; Badran et al. 2014; Papadimitriou et al. 2020). Participants further suggested that the relevance and impact of malocclusion vary depending on broader contexts, such as societal values, the availability of care, and a nation’s wealth. This again highlights that MNOC cannot be a purely medical concept and that the voices of all key actors should be considered given that MNOC may not be amenable to a static definition.

### Where Does This Leave Us?

It appears that, on its own, MNOC in its current form is ill-suited to do any heavy policy lifting. Not only is there limited evidence linking normative measurements of malocclusion to health and functioning, but the notion of MNOC cutoffs as objective thresholds is also questionable, given their basis in professional consensus rather than empirical absolutes. Moreover, psychosocial factors are often overlooked when classifying cases as MN, which is inconsistent with today’s biopsychosocial model of health. It may be more helpful to conceptualize MNOC using an onion model to stratify cases, as has been described for “essential oral health care” more broadly (Benzian et al. 2021). The innermost layer would include cases for which there is virtually no controversy as to whether the patient would benefit from treatment. The middle layer would comprise cases that are less clear-cut but have features of the innermost layer. The outer layer would consist of cases that, if treated, would lead to the least tangible benefits to the patient. Nonetheless, this still leaves us with the practical issue of how to assign an orthodontic case to 1 of the 3 layers; this challenge is addressed via the following recommendations.

### Recommendations

Establishing a precise definition or rigid boundaries for MNOC may offer limited practical value given the moral and conceptual controversies inherent in determining normative needs in health care. As a result, some analysts have advocated for deliberative approaches to priority setting and resource allocation (Daniels and Sabin 1997). One such approach could fuse orthodontic indices, oral health–related quality-of-life questionnaires, and meaningful deliberation between key actors. The process may look as follows:

1. Create a committee with representatives from all OC key actor groups.
2. Deliberate based on distinct interests and values of these key actors to select:
  a. One index to quantify malocclusion based on occlusal and cephalometric measurements.
  b. One questionnaire to measure the impacts of malocclusion on patients’ oral health–related quality of life.
  c. Eligible populations for funding of MNOC.

3. Screen eligible individuals using 2a and 2b to select those with greatest need:
  a. Automatically approve individuals with high need in both categories.
  b. Deliberate over cases showing high need in one category but not the other.

4. Select high-need individuals to receive funding for MNOC based on available resources.

Deliberating over need under resource constraints can ensure that even when there is disagreement among key actors, decisions are more likely to be reasonable and fair (Daniels and Sabin 1997). By viewing MNOC as a deliberative process that incorporates the viewpoints of all involved, access to care can be facilitated for those likely to experience tangible benefits to their health and/or quality of life.

### Limitations

The background and demographics of participants may have influenced the results of this study. All participants were from high-income nations, and most were male, which may have shaped their perspectives on MNOC, as dental needs and beauty standards can vary greatly across regions and socioeconomic contexts. In addition, the public was underrepresented, which may have led to a skewed understanding of the factors deemed most important in determining MNOC. The perspectives of patients may differ significantly from those of professionals, potentially highlighting factors not captured in this study. Since this study demonstrated that MNOC is a fluid concept dependent on cultural values and contextual factors, the findings from this specific group of respondents may not be universally applicable. Furthermore, the use of video recordings may have introduced bias, such as the Hawthorne effect, in which participants might have altered their behavior due to being observed or concerns about anonymity that could have limited how openly they responded. Nonetheless, it is believed the fundamental principles underpinning MNOC—particularly the key factors for consideration—are likely to remain relevant, even as their interpretation and application vary across settings and contexts.

## Conclusion

Governments and other key actors should focus on clarifying their interpretation of MNOC as they develop strategies for including OC within UHC, if that is an identified goal. This study outlines the range of dental, medical, psychosocial, and funding factors that can inform this effort. Nevertheless, given that MNOC is a fluid concept shaped by cultural values and contextual factors, it may be best for the approach to determining which cases qualify as MN to evolve into a deliberative process that incorporates diverse key actor input. This would enable transparent and fair coverage decisions, securing care for those with demonstrated need in the context of health, well-being, and quality of life.

## Author Contributions

D. Richmond, contributed to conception, design, data analysis, drafted and critically revised the manuscript; H. Benzian, J. Daskalogiannakis, A. Holden, C. Quiñonez, contributed to conception, design, critically revised the manuscript. All authors gave final approval and agree to be accountable for all aspects of the work.
